# Red-complex bacteria: immunological background leading to the development of head and neck cancers

**DOI:** 10.3389/fimmu.2026.1804268

**Published:** 2026-04-01

**Authors:** Karolina Kaźmierczak-Siedlecka, Robert Kucharski, Adam Kosiński, Luigi Marano, Wojciech Makarewicz, Leszek Kalinowski

**Affiliations:** 1 Department of Medical Laboratory Diagnostics – Fahrenheit Biobank BBMRI.pl, Medical University of Gdansk, Gdansk, Poland; 2 Neodentica Dentistry Center, Gdansk, Poland; 3 Department of Clinical Anatomy, Medical University of Gdansk, Gdansk, Poland; 4 Academy of Applied Medical and Social Sciences, Elblag, Poland; 5 2nd Division of Radiology, Medical University of Gdansk, Gdansk, Poland; 6 BioTechMed Center, Department of Mechanics of Materials and Structures, Gdansk University of Technology, Gdansk, Poland

**Keywords:** head and neck cancers, immunology, Porphyromonas gingivalis, Tannerella forsythia, Treponema denticola, virulence factors

## Abstract

Oral microbiome imbalance is involved in the development of head and neck cancers (HNCs). There is a group of oral pathogens, such as *Porphyromonas gingivalis*, *Tannerella forsythia*, and *Treponema denticola*, that creates red-complex. Currently, the oral pathogens’ role in the pathogenesis of periodontitis is well-described; nevertheless, data regarding the link between HNCs and periopathogens are still limited, especially considering *T. forsythia* and *T. denticola*. These microbes utilise various virulence factors to promote the carcinogenesis process, affecting the immunological background. This paper presents modern insights into the role of red-complex bacteria in the development of HNCs, concentrating on the immunological aspects.

## 1 Introduction

Malignancies arising from the nasal/oral cavity, pharynx, larynx, paranasal sinuses, and salivary glands are classified as head and neck cancers (HNCs) (1). They are ranked as the sixth most common malignant tumour globally. The GLOBOCAN 2020 database indicated that there are approximately 930,000 new cases and 467,000 deaths per year (2). Approximately 90% of cases are diagnosed as head and neck squamous cell carcinoma (HNSCC), whereas approximately 70%–80% of cases are found at stage III/IV (2). In addition to commonly known risk factors, oral microbiome imbalance also contributes to the development of HNCs. There is no consistent signature of oral dysbiosis in HNC patients; however, the abundance of some microbes, such as *Fusobacterium*, *Leptotrichia*, *Treponema*, *Porphyromonas gingivalis*, Enterobacterales, *Pseudomonas*, *Prevotella*, Bacteroidetes, *Haemophilus*, and *Veillonella*, was noted (3). Red-complex pathogens are a group consisting of *P. gingivalis*, *Tannerella forsythia*, and *Treponema denticola* (4). Periodontopathogens are linked to the development of different tumours, including HNCs; however, molecular/immunological processes are not well defined (5, 6).

Considering epidemiology data and the fact that HNCs are a heterogeneous group of tumours and often diagnosed at an advanced stage, there is a need to detect underlying mechanisms, inhibit them, and detect cancer in the early phase. The understanding of immunological pathways is crucial in this context. One of the examples is the role of neutrophilic cells, which belong to the most abundant immune populations in the HNC tumour microenvironment (TME), being significant mediators of local immunosuppression. They are phenotyped as CD11b+CD14−CD15+/CD66b+. Notably, neutrophilic cells act in the following ways: 1) promote tumour cell growth, 2) inhibit cancer cell senescence through the expression of interleukin-1 receptor antagonist (IL-1Ra), and 3) stimulate angiogenesis, affecting the secretion of vascular endothelial growth factor (VEGF), prokineticin-2 (BV8), and matrix metalloproteinase-9 (MMP-9). Considering clinical practice, it should be emphasised that the neutrophil-to-lymphocyte ratio (NLR) is a biomarker of systemic inflammation (7, 8). Recently, Kao et al. reported that high-NLR patients (who underwent HNC surgery) had increased odds of 30-day mortality (p < 0.001) compared to subjects with low NLR (8). Neutrophilic cells can also present antitumour activity. It confirms the complexity of action and involvement in cancer-related pathways in different ways (7).

Insights into the TME in the case of oral squamous cell carcinoma (OSCC), which is a highly aggressive tumour of the oral cavity, reveal the complexity of its immunological components regarding T lymphocytes, fibroblasts, neutrophils, myeloid-derived suppressor cells (MDSCs), tumour-associated macrophages (TAMs; both M1 and M2), and mediators (cytokines) with their signalling role. Of note, CD4+ T lymphocytes promote nodal metastasis and activate regulatory T cells and T helper cells. CD8+ lymphocytes are associated with tumour inflammation and cytotoxicity. In the context of cancer cells, the polarisation of TAMs is crucial for the obtained macrophage activities. The polarisation of M1 TAMs and M2 TAMs is driven by different mediators [M1: lipopolysaccharide (LPS), IL-12, IL-18, and tumour necrosis factor alpha (TNF-α); M2: IL-4, IL-13, and transforming growth factor beta (TGF-β)] that cause other effects (9). Different subsets of immune cells included in the TME are strongly linked to tumour progression and response to treatment (10).

Due to the fact that previous data mainly described the role of *P. gingivalis* in periodontitis and cancer development, the current paper additionally presents other microorganisms belonging to the red-complex pathogens (i.e., *T. forsythia* and *T. denticola*) with their immunological mechanisms of action contributing to HNC occurrence.

## 2 P. gingivalis

*P. gingivalis* is a Gram-negative oral anaerobic bacterium assessed as a major pathogen in periodontitis (11, 12). It is also linked to distant tumours, such as pancreatic cancer (by promoting pancreatic tumourigenesis through, among others, neutrophil elastase), being considered a microbial biomarker that allows for its detection in the early stage (13, 14) and gastric cancer (by affecting both gastric carcinogenesis and progression) (15). *P. gingivalis* utilises virulence factors to override innate and adaptive immune responses. By suppressing adaptive immunity, *P. gingivalis* is able to exist in the host tissues, resulting in a persistent inflammatory response (16). The virulence factors associated with *P. gingivalis* are as follows: cysteine proteases (gingipains), fimbriae (short Mfa pili and long Type V pili), nucleoside diphosphate kinase (NDK), and LPS (5, 17). Gingipains induce the release of other pro-inflammatory mediators. Similarly, LPS increases the secretion of pro-inflammatory cytokines (15). Pattern recognition receptors (PRRs), such as toll-like receptors (TLRs) and NOD-like receptors (NLRs), are responsible for the recognition of *P. gingivalis* and its virulence factors. Nevertheless, this bacterium is able to avoid recognition and additionally inhibit the autophagic pathway. It affects the host’s innate immune response, causing loss and damage of periodontal ligaments and alveolar bone (18). Moreover, interspecies interactions can be mediated by *P. gingivalis* vesicles, contributing to the coaggregation of *T. denticola* and *Lachnoanaerobaculum* (19). Some evidence also suggests that co-infection caused by *P. gingivalis* and human cytomegalovirus (which may act synergistically) creates a favourable environment for the development of oral cancer (20). Recently, in 2025, it was demonstrated that oral tumourigenesis is promoted by *P. gingivalis* outer membrane vesicles (OMVs) by inhibiting innate immune surveillance (21). The mechanism is based on the ability of *P. gingivalis* OMVs to suppress cyclic GMP-AMP synthase (cGAS)–stimulator of interferon genes (STING)–IFN β innate immune signalling, resulting in the impairment of antitumour immunity (21).

In a systematic review of 17 articles (14 *in vitro* and three with animal models), it was shown that *P. gingivalis* (strains ATCC 33277, 381, and W83) is related to the development of OSCC (22). Its role has been observed in three different phases: 1) epithelial–mesenchymal transition of malignant cells, 2) neoplastic proliferation, and 3) tumour invasion. Recently, in 2025, the differential gene expression profile in human gingival keratinocytes treated with *P. gingivalis* and its link to HNSCC was analysed (23). Notably, three genes, such as *FST*, *VRK3*, and *SGK1*, were overexpressed in these patients, whereas the *FST* gene was additionally associated with poor prognosis. Katz et al. reported that the abundance of *P. gingivalis* was detected in carcinoma samples (n = 10 gingival squamous cell carcinoma, paraffin-embedded samples, and immunohistochemical staining) compared to normal gingival tissues (24).

One of the immunological mechanisms by which *P. gingivalis* is involved in OSCC development is based on the protection of cancer from macrophage attack. It was demonstrated in the Liu et al. study, which assessed the influence of *P. gingivalis* on the phagocytosis of Cal-27 cells by bone marrow-derived macrophages *in vitro* and evaluated the effect of this bacterium on OSCC growth and the polarisation of tumour-associated macrophages *in vivo* (25). Of note, in 2025, it was demonstrated that *P. gingivalis* is positively correlated with cancer stem cell marker expression in human OSCC specimens (26). The expression of stearoyl-CoA desaturase 1 (SCD1) is upregulated by *P. gingivalis*, which increases the synthesis of lipid in OSCC cells. Moreover, this upregulation is linked to the expression of KLF5 and NOD1 signalling. Therefore, the NOD1/KLF5 axis may play a significant role in regulating SCD1 expression.

## 3 T. forsythia

*T. forsythia* (Gram-negative oral pathogen) is affiliated with the Bacteroidetes phylum (*Cytophaga*–*Bacteroides* family, genus *Tannerella*), being a key member of the oral biofilm consortium (27, 28). It forms synergistic biofilm with *Fusobacterium nucleatum in vitro*. This co-infection increases inflammatory alveolar bone loss (29). The group of virulence factors of *T. forsythia* includes enzymes (sialidase and proteases), cell surface proteins [2D crystalline cell surface (S-) layer, glycosylation, and cell surface antigen BspA], OMVs, and rough-type LPS (27). These virulence factors are able to disturb epithelial cells and stimulate host immune responses. Consequently, it leads to the process in which *T. forsythia* enters the circulatory system and affects distal organs and the aorta with important immune cells (CD3+ T cells and IL4R-positive CD3+ Th2 T cells) (30). The results of the study conducted by Jung et al. suggest that *T. forsythia* GroEL (molecular chaperone homologous to human heat-shock protein 60) may be a novel virulence factor contributing to the inflammatory bone resorption related to *T. forsythia* (31). Of note, the pathogenicity of *T. forsythia* species could be strain-dependent. The ability of two *T. forsythia* clinical isolates (UB4 and UB20) to activate macrophages was investigated in the Chinthamani et al. study (32). UB20 is a more potent inducer of the expression of CXCL10 (chemokine protein IP-10) than the abovementioned UB4, as well as the laboratory-adapted strain ATCC 43037. Settem et al. reported that this laboratory strain of *T. forsythia* is able to scavenge peptidoglycan and affect the innate immunity of oral epithelium through the dampening of nucleotide-binding oligomerisation domain (NOD) activation. Recently, in 2025, a systematic review (regarding 131 articles) reported that *T. forsythia*, *Fusobacterium*, and the above-described *P. gingivalis* are increased in oropharyngeal squamous cell carcinoma (33).

## 4 T. denticola

Major surface protein (Msp) and surface protease dentilisin are virulence factors of *T. denticola* (34, 35). Nevertheless, chymotrypsin-like proteinase (CTLP) is the major virulence factor of this bacterium. In the Listyarifah et al. (36) study, this virulence factor was detected in 95% of mobile tongue squamous cell carcinoma cases. Notably, the analysis of the immunopositivity of *T. denticola*-CTLP revealed its correlation with invasion depth, diameter of tumour, and the expression of c-Myc, as well as TLR-7 and TLR-9. CTLP may contribute to carcinogenesis. Its involvement in the carcinogenic process is observed through immunomodulation in orodigestive tumours. *T. denticola*-CTLP is able to convert pro-MMP-8 and pro-MMP-9 into their active forms (37). Of note, MMPs are key enzymes responsible for the degradation of extracellular matrix proteins. Recently, in 2025, it was shown that radiotherapy used to treat HNCs increases the activities of MMP-8 and MMP-9, which consequently leads to the progression of periodontitis (38).

Considering oropharyngeal squamous cell carcinoma, *T. denticola*-CTLP was found in 81% of cases, mainly in human papillomavirus (HPV)-negative tumours (39). Lee et al. (40) reported that *T. denticola* is the most abundant in OSCC subjects compared to the stomatitis and healthy control groups. Additionally, the amount of *T. denticola* was positively related to *Lactobacillus casei* (p < 0.001) and *Candida albicans* (p = 0.008), confirming the correlations between them. *T. denticola* is implicated in the pathogenesis of OSCC by promoting the proliferation of OSCC cells, and the background mechanism is associated with the activation of the intracellular TGF-β signalling pathway (41). In another study (42), it was demonstrated that the incidence of OSCC is positively associated with the increased expression of TLR-4/9 mRNA/protein, bacterial infection, and gingival inflammation/gingival recession (GIGR). The study group comprised 120 OSCC subjects, among whom 85 cases had *P. gingivalis*, *F. nucleatum*, *T. denticola*, and GIGR.

**Figure 1:**
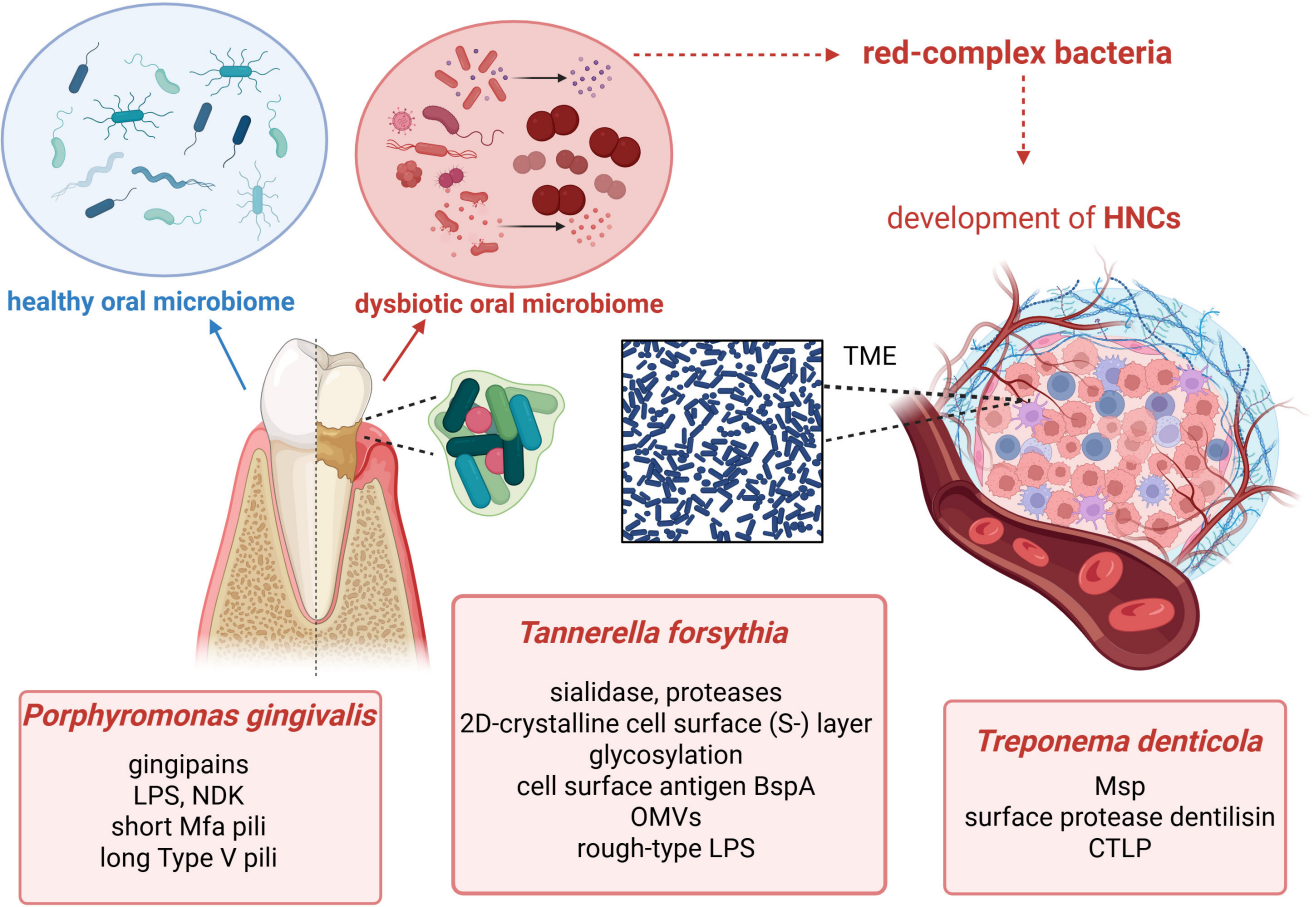
Dysbiotic oral microbiome signature is not consistent among HNC patients. Its imbalance contributes to the development of tumours in oral cavity/neck. Red-complex bacteria, such as *Porphyromonas gingivalis*, *Tannerella forsythia*, and *Treponema denticola*, are mainly known as oral pathogens involved in periodontitis; however, they can also participate in pathogenesis of HNCs. These bacteria utilise multiple virulence factors, allowing them to modulate molecular and immunological pathways. This figure was created using BioRender.com. LPS, lipopolysaccharide; NDK, nucleoside diphosphate kinase; OMVs, outer membrane vesicles; Msp, major surface protein; CTLP, chymotrypsin-like proteinase; TME, tumour microenvironment; HNC, head and neck cancer.

The summary of virulence factors that red-complex pathogens utilise is presented in **Figure 1**.

## 5 Discussion and conclusions

Dysbiotic oral pathogens belonging to the red-complex bacteria group participate in the development of HNCs in different ways. They utilise virulence factors to manipulate the functions of the immune system and regulate immune response through multiple mechanisms. In addition to their typical activity observed as an interaction between microbes, immune cells, and cancer cells, there are also interspecies coaggregation and co-infection (with other bacteria, fungi, and viruses). These interactions should be considered during the identification of molecular/immunological pathways involved in the carcinogenesis process.

There is a strong link between dental problems and HNCs, particularly if taking into consideration oral pathologies associated with microorganisms. The risk of HNCs is increased in individuals with periodontitis (43, 44). In a case–control study (45), it was demonstrated that patients with periodontitis are 3.7 times more likely to develop OSCC compared to subjects without this disease. Considering both TNM and the location of the tumour, the differences in periodontitis were not statistically significant (p > 0.05). However, this relationship was highlighted among the following features: men, age > 60 years, and more tooth loss. Periodontitis-related bacteria, such as *P. gingivalis* and *F. nucleatum*, are 600 times more frequently found in OSCC compared to normal tissues (43, 46). Nevertheless, there are some data that indicate that microorganisms involved in the development/progression of periodontitis are not directly connected to HNC occurrence. This critical point of view is supported by results provided by, for instance, Kwak et al. (47) in a study highlighting the problem of HNSCC and periodontitis-associated bacteria. It has been reported that periodontal pathogens belonging to the red/orange complex are associated with a moderately increased risk of HNSCC. Therefore, HNC incidence should rather be considered in the context of a multifactorial background and co-existence of crucial factors than as periodontitis-associated microorganisms alone. Moreover, the signature of oral microbiome imbalance in patients with HNCs is not consistent. Nevertheless, high loads of some oral pathogens’ DNA are often detected in these cases. The establishment of oral microbial biomarkers allowing for the screening and selection of subjects with a high risk of HNC development or their detection in the early stage (specifically stage I under TNM classification) is challenging. Several factors should be considered, such as the method of microbiome analysis (16S and shallow shotgun), the similar surface of the oral swab taken, the preparation of the patient for an oral swab, oral hygiene, and the presence of other diseases (mainly periodontal-related pathologies). The cooperation between oncologists and dentists is crucial in this context.

There are some limitations of this paper associated with the construction of a mini-review. First of all, it used a non-systematic search strategy, which affected the strength of evidence. Thus, it provided the point of view based on the heterogeneity of included studies with not well-established inclusion criteria. For instance, there are available different methods of microbiome analysis, such as shallow shotgun or 16S rRNA (with additionally various regions, i.e., V3, V4, or V1–V9), influencing the level of obtained results. Additionally, the signature of the oral microbiome and the selection of particular bacteria are strongly connected with sample types (saliva, oral rinse, and oral mucosal swab). Of note, data based on one technique of microbiome analysis and one sample type can provide more details and a more precise signature. Moreover, this article discusses overall HNCs despite the fact that it is a heterogeneous group of tumours with different localisations (oral cavity/neck); however, this classification is approved and commonly used in clinical practice. Despite the fact that mostly HNCs are assessed as squamous cell carcinoma (approximately 90% of cases), the selection of tumours with other histo-pathological diagnoses could give more precise data, similar to the analysis of cases according to the stage.

To summarize, oral microbiome imbalance, especially with high loads of oral pathogens’ genetic materials, is involved in the pathogenesis of HNCs. Red-complex bacteria previously considered mainly in the context of periodontitis are able to participate in the carcinogenesis of tumours located in the oral cavity/neck. They utilise virulence factors alone or together with other microorganisms either synergistically or antagonistically. Immunological mechanisms linking oral pathologies, dysbiotic oral microbial signature, and tumourigenesis of HNCs are still not well discussed; however, they exist and provide a promising strategy as targets for personalised medicine. Therefore, it is recommended to analyse the oral microbiome to establish an oral microbial signature and introduce a precision approach.
